# Using Artificial Intelligence Assisted Learning Technology on Augmented Reality-Based Manufacture Workflow

**DOI:** 10.3389/fpsyg.2022.859324

**Published:** 2022-06-29

**Authors:** Mingchao Li, Yuqiang Chen

**Affiliations:** School of Artificial Intelligence, Dongguan Polytechnic, Dongguan, China

**Keywords:** artificial intelligence assisted learning, workflow management, augmented reality, intelligent manufacturing, Industry 4.0

## Abstract

The manufacturing process is defined by the synchronous matching and mutual support of the event logic and the task context, so that the work task can be completed perfectly, by executing each step of the manufacturing process. However, during the manufacturing process of the traditional production environment, on-site personnel are often faced with the situation that on-site advice is required, due to a lack of experience or knowledge. Therefore, the function of the manufacturing process should be more closely connected with the workers and tasks. To improve the manufacturing efficiency and reduce the error rate, this research proposes a set of manufacturing work knowledge frameworks, to integrate the intelligent assisted learning system into the manufacturing process. Through Augmented Reality (AR) technology, object recognition technology is used to identify the components within the line of sight, and the assembly steps are presented visually. During the manufacturing process, the system can still feedback to the user in animation, so as to achieve the function equivalent to on-the-spot guidance and assistance when a particular problem is solved by a specialist. Research experiments show that the operation of this intelligent assisted learning interface can more quickly recognize how the manufacturing process works and can solve problems, which greatly resolves the issue of personnel with insufficient experience and knowledge.

## Introduction

In today's industrial environment, manufacturing process assembly often relies on the dexterity of human beings. These manual methods and principles are accumulated from many experiences but are gradually losing their heritage as technology advances. Despite the development of digitally controlled production equipment in Industry 4, the technology has become quite mature, and many companies use robotic arms to assemble parts. However, the assembly of small parts is still a problem, due to the economic benefits of relying on tactile sensations and other factors executed by hand. When the manufacturing production reaches a certain scale and the work reaches a certain level of complexity, the number of people and the level of technology used will increase, but the ability of people to conduct the work will vary, resulting in variable levels of experience. Most of the assembly parts in the manufacturing process have a straightforward assembly sequence and operation procedure, therefore, almost every assembly action can be carried out by the Standard Operation Procedure (SOP). The SOP takes into consideration the overall connection relationship of the operation details of an event, allows for systematic planning, can confirm the satisfaction of cause and effect, and through quantitative management can control its process, resulting in each operator achieving a considerable degree of homogeneous quality and efficiency. This solves the problem of unequal human capacity (Calonder et al., 2010; Bochkovskiy et al., 2020).

The purpose of establishing the manufacturing process with the SOP is to standardize the work, therefore, the design of the manufacturing process in conjunction with the SOP is very important. In today's society, the SOP manufacturing process has gradually developed from text, images, and a process control mechanism, to ensure awareness and understanding at each stage. However, since the interpretation methods and knowledge backgrounds of manufacturing process personnel may not be the same, the following situations may also occur during on-site work, for example, the manufacturing process is not able to respond in real-time, and the psychological pressure capacity is insufficient. Different experiences lead to cognition and insufficient operation error, several factors that affect the quality and efficiency of the operation. If the SOP of the manufacturing process can be presented in a more intuitive and understandable way, and then tracked through process control, it will greatly improve users' understanding, reducing errors and risks. The SOP of the manufacturing process can help operators understand the details of manufacturing and assembly tasks, but to date, most of the professional background knowledge and practical experience is still in the hands of masters. The practical way to pass on knowledge in today's industrial environment is to formulate standard operating procedures and then gradually revise the SOP of the manufacturing process according to the conditions. This means that the SOP of operating procedures can maintain a certain quality but may not be perfect, and cloud notes can supplement additional knowledge for personnel, but the personnel may vary. There must be sufficient time to read the procedures, but even so, it is difficult to achieve good cognition quickly. Therefore, this article will redefine the type of the SOP of the manufacturing process in an intuitive way, so that personnel can ask language questions about the program content they do not understand, and allow the system knowledge base to answer them, which will increase the personnel's understanding of SOPs, The study also aims to supplement the knowledge of the manufacturing process in real-time in a “learning by doing” way.

Among today's technology are many increasingly mature technologies, such as AR technology and related application products like HoloLens2HoloLens22, issued by Microsoft, Vive by HTC, and CATIA equipment and software. Many companies are now developing the in-depth application of such equipment. For example, HoloLens2HoloLens22 can use AR technology to create images in real space, and it can have spatial cognition, which is a great help to the development of practical projects (Fiorentino et al., 2014; Wu et al., 2016b). Vive can be integrated into the viewing of 3D virtual space to simulate a real operating environment, so as to improve proficiency; CATIA Composer can operate 3D models with special effects, such as moving, rotating, scaling, cutting, and translucency, which is not easy to achieve in reality through screen operations. From the factory environment to the assembly screws, field personnel can understand the equipment structure more clearly, so that the skilled use of these equipment software packages can substantially reduce the difficulty of on-site operation. Consequently, this application trend has been established in the current industry. Using Industry 4.0 smart manufacturing technology, in addition to gradually replacing repetitive human operations with numerical control equipment, manufacturers also need to use system intelligence to connect all the trivial matters in the production process (Faccio et al., 2019; Zheng et al., 2020). As manufacturing process jobs become increasingly proficient with the use of intelligent machines, people will become increasingly unfamiliar with their jobs. Gradually, no one on-site will be able to understand all the work at all times. However, in the case of manufacturers, who have introduced the digital smart manufacturing process, SOP, at a particular stage of the standard operation, AR technology can greatly reduce the difficulty of work. If there is a lack of knowledge, through the system knowledge base, knowledge can be added immediately. Therefore, to create a highly intuitive standard operation program for the manufacturing process and build a systematic knowledge base to provide interactive questions and answers, the specific research purposed an advantage can be summarized as follows:

1. To use AR technology in the wearable device, HoloLens2HoloLens22, to perform intuitive virtual interactive operations.
2. To use object recognition technology to closely fit the image outline of the 3D model to the real components in AR space.
3. To use Automatic Speech Recognition (ASR) to parse question text.
4. To use Natural Language Processing (NLP) to understand the user's semantics and to answer based on the basic system knowledge base.

The limitations:

1. The object recognition technology used in this study is limited to the feature algorithm in the 2D recognition element, using 2D recognition technology to make the model fit the real object, and the ambient light is sufficient.
2. Only the feasible integration technologies of HoloLens2HoloLens22 and unity software are discussed, and the technologies required for other AR devices are not within the scope of this study.
3. For the reading comprehension task in this study, only the BERT pre-training model is used, and other pre-training models are not within the scope of this study (Devlin et al., 2018; Chen et al., 2019a).

The contribution of this article, through Augmented Reality (AR) technology, object recognition technology is used to identify the components within the line of sight, and the assembly steps are presented visually. During the manufacturing process, the system can still feedback to the user in animation. To achieve the function equivalent to the on-the-spot guidance and assistance to solve the problem by a special person. Research experiments show that the operation of this intelligent assisted learning interface can more quickly recognize the manufacturing process work and solve problems, which greatly solves the situation of personnel with insufficient experience and knowledge.

This research is divided into five sections, the first section includes the introduction, the research background, research motivation, research purpose, and research limitation. The second section is the literature review, discussing the literature on AR, object recognition, NLP, and related AR applications, as well as considering the application and principles of certain literature, so as to have a deeper understanding of its application fields and functions. The third section is the research method, which introduces the 3D model, feature algorithm, NLP, and system core construction. Section four comprises the implementation and results in comparison and section five contains the conclusion and aspects relating to future work.

## Literature Review

This research is based on AR and constructs a system for ASR.

### AR

**Figure 1:**
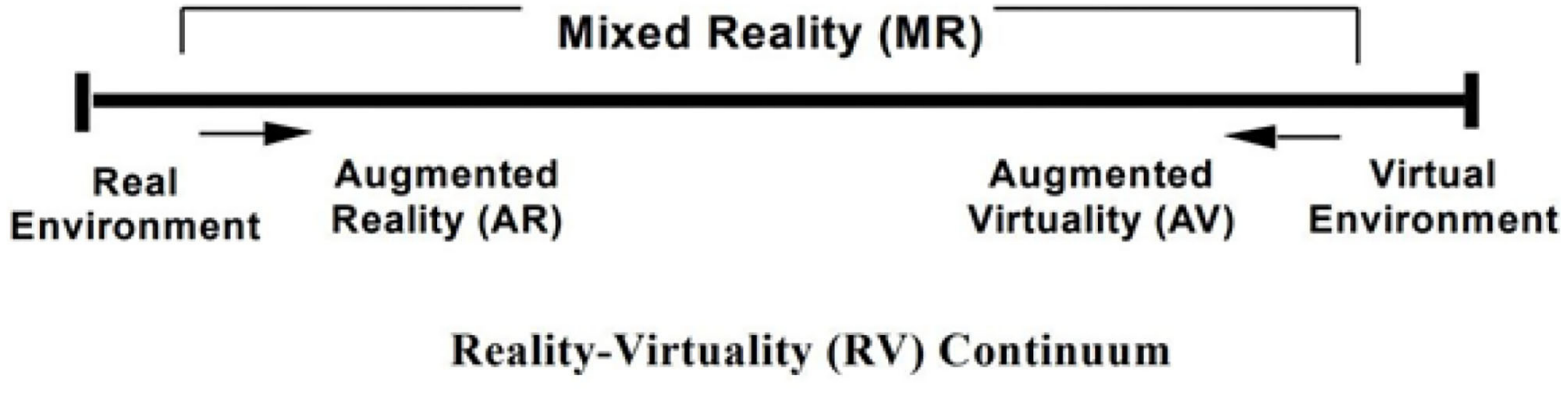
Real-virtual continuity system.

The real and virtual are located at both ends of the system, that which is close to the real is called AR, and that closest to the virtual is called Augmented Virtuality (AV). The augmented virtual world is referred to as Mixed Reality (MR). One of the aspects that are easily confused is the Virtual Reality (VR) technology, as shown in Figure 1. The biggest difference between VR and AR is the environment. All objects, scenes, characters, etc. in VR are virtual and the interaction is limited to the virtual space, so the former is mostly used to simulate the real environment, while AR places virtual objects in the real environment. This article has a wide range of uses and a more specific definition of AR. The study believes that AR needs to have the following three characteristics: combining virtual objects with reality, instant interaction, and 3D.

**Figure 2:**
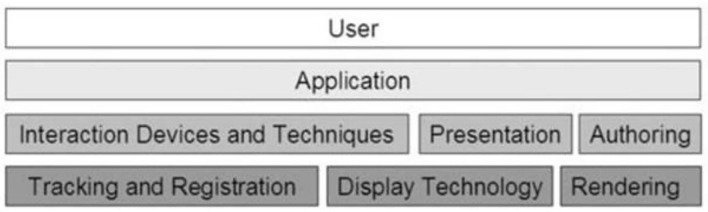
The basics of augmented reality.

The application basis of AR, as shown in Figure 2, consists of various modules, including users, applications, display technology, rendering technology, and interaction, which are all essential elements.

#### Microsoft HoloLens2HoloLens22

The HoloLens2HoloLens22 has a depth sensor, which can obtain the depth information of the environment and then achieve the reconstruction of the real space through Simultaneous Localization and Mapping (SLAM) technology; the image output adopts the optical penetration imaging principle. Through a piece of semi-reflective and semi-transparent glass, the light can penetrate the glass for the eye to see and then passes through the Liquid Crystal on Silicon projection technology, which projects the image onto the semi-transparent glass, so as to achieve an overlap of the real picture and the virtual picture, making it visible to the naked eye. Although the first-generation HoloLens2HoloLens22 was powerful, it still had many shortcomings, such as uncomfortable wearing, a narrow field of vision, and short battery life, while the second-generation HoloLens2HoloLens22 had greatly improved specifications in all aspects, and also added new features, such as eye-tracking.

### Object Recognition

Object recognition identifies pre-defined objects from images. There are many methods, such as deep learning and feature algorithm. Computer vision is an esoteric branch of the field, and many people are still researching this technology. Object recognition is a general term to describe a collection of related computer vision tasks that involve identifying objects in digital photographs. Image classification involves predicting the class of one object in an image. Object localization refers to identifying the location of one or more objects in an image and drawing a bounding box around their extent. Object detection combines these two tasks and localizes and classifies one or more objects in an image.

#### YOLOv4

You Only Look Once (YOLO) technology is one of the most classic methods in 2D recognition AI algorithms. The v4 version of YOLO is more accurate and faster than many modern detection methods and is more achievable. There are a large number of features that are said to improve Convolutional Neural Network (CNN) accuracy. Practical testing of combinations of such features on large datasets and the theoretical justification of the result are required. Some features operate on certain models exclusively and for certain problems exclusively, or only for small-scale datasets; other features, such as batch-normalization and residual-connections, are applicable to the majority of models, tasks and datasets. Real-time detection, e.g., YO LOv4 has been widely used in various fields, such as medicine, industry, education, and government agencies. The practical application can be used for the identification of many dynamic objects, so as to achieve various security or management purposes (Chen et al., 2019b; Huang et al., 2019).

#### PoseCNN

PoseCNN is a 6D pose estimation method based on deep learning. The end-to-end 6D pose estimation model by Xiang et al. (2017), which can be efficient in dealing with the occlusion problem is introduced, namely, a new pose estimation loss function is known as ShapeMacth-Loss; a large-scale RGB D image dataset, YCB-Video, is provided for 3D estimation, including 21 objects, 92 videos, and 133,827 pictures. PoseCNN uses RGB as input and uses 13 convolutional layers and four maximum pooling layers to extract features, predict each object and calculate its center, perform semantic segmentation for each known object and crop the original image and point cloud. The Hough voting layer is used to predict the target bounding box, and the two region pooling layers are combined and sent to three fully connected layers to finally output the 3D rotation represented by the quadruple (Chen et al., 2019b; Ye et al., 2019, 2021; Fu, 2022).

### NLP

Natural language processing (NLP) has become increasingly mature in the field of deep learning, and many companies use its technology to develop their own personal assistants, such as Apple's Sifi, Google Assistant, and Microsoft's Cortana. The development lies in understanding human language and making different responses according to different tasks. NLP is roughly divided into Natural Language Understanding (NLU) and Natural Language Generation (NLG), which involves understanding human language, and the robot itself generates text that humans can understand. NLU can help the system understand the text input (Liu et al., 2014; Lin et al., 2020). The smallest unit is a word, multiple words form a sentence and multiple sentences can form an article, indicating that the system must first be able to identify words, then uses the relationship between words to understand the entire sentence; word embedding is a more common training method. The words themselves are marked with vectors of different dimensions and after a great deal of training, an effective model can be trained. The vector distance between words represents their relationship, for example, the vector distance of prince and princess will be roughly equal to husband and wife (Huang et al., 2017; Chen et al., 2020b). The most popular algorithm model for researchers is Bidirectional Encoder Representations from Transformers (BERT), which is a set of languages open sourced by Google and based on the Transformer framework proposed by Devlin et al. (2018). There are also many models, such as Word2Vec and GPT.

#### BERT

Bidirectional Encoder Representations from Transformers (BERT) is Google's use of unsupervised methods to train data, using a large amount of unlabelled text for training. BERT is competent in all aspects of NLP tasks, and its performance is extremely high. There are a large number of research articles based on BERT to improve efficiency, using language models, as they have various benefits:

1. Unsupervised learning means that the amount of data is huge, and there is no need to label the data. All texts on the Internet are datasets. BERT pre-training uses 3.3 billion words, including Wikipedia and BooksCorpus.
2. A good language model can learn to deconstruct grammar, and interpret semantics and can be more efficiently applied to downstream tasks through feature extraction to improve its performance.
3. BERT reduces the architectural cost of different NLP tasks. Two-stage transfer learning is very popular in the NLP field. Firstly, a language model pre-training method is used to train a model with a certain degree of understanding of natural language, then the model is used for feature extraction or to fine-tune downstream tasks. Since the pre-training cost of BERT is too high, the model that has been trained by BERT itself is usually used for downstream supervised tasks. The third aforementioned item stated that the use of BERT can reduce the cost of different NLP task architectures (Chen et al., 2015; Huang et al., 2015). Different NLP tasks have different model architectures, but if BERT is used, the pre-training architecture is exactly the same as the general downstream task architecture, which can also greatly reduce the architectural cost of designing the model.

#### Slot-Filling and Intent

**Figure 3:**
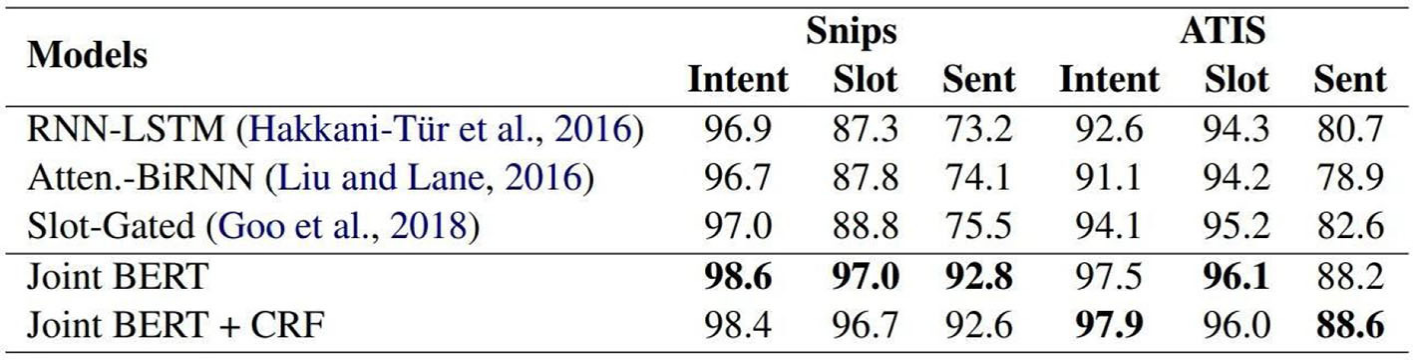
F1 Score on both datasets.

Intent refers to the intent or field that the user wishes to express in one sentence, also known as slot filling. When a computer wants to analyze a sentence, it needs to grasp the key points of the sentence one by one. In this process, each word needs to be classified in terms of its part of speech: noun, verb, adjective, etc. At this stage, neural networks can be used to capture the part-of-speech and the meaning of each word. Usually, when sentences are used, memory networks are used (Chen et al., 2020a, 2021). The more common is the Long Short-Term Memory (LSTM) which is a Recurrent Neural Network (RNN); LSTM is also used in Google Translate (Wu et al., 2016b). As mentioned above, intent analysis and slot filling basically complement one other and usually act on task-based chatbots. After judging the user's intention, the slot is filled in the closed field to achieve the function of executing the task; the slot has time, origin, and destination (Rosten and Drummond, 2006). Through extensive neural network training, the field of each piece of vocabulary can be automatically captured and the intention analysis and slot filling can be trained together since the input is the same sentence, the only difference is the label. BERT is used for joint learning and has obtained better results than the other models on the two training sets of Snips and ATIS. As shown in Figure 3, Snips and ATIS are a large training set for both intent and slot filling tasks.

#### Seq2Seq

**Figure 4:**
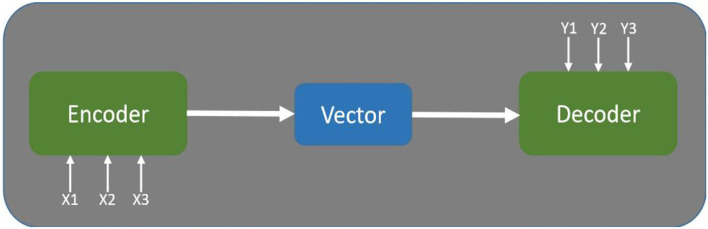
Encoder-decoder architecture.

Seq2Seq is the abbreviation of sequence-to-sequence, which means inputting one sequence and outputting another sequence. This is the architecture of Encoder-Decoder as Figure 4 and the length of the input and output is variable. The related applications, including Google machine translation (Sutskever et al., 2014) and Google speech recognition (Prabhavalkar et al., 2017), are very versatile.

#### Image Caption

The image caption is a technology that can generate textual descriptions for images, most of which belong to the Encoder-Decoder framework, therefore, Google (Vinyals et al., 2015) proposed an image captioning model, which combines machine vision and machine translation to generate sentences describing images. Xu et al. (2015) introduced an attention mechanism based on image description. Image captioning is the task of providing a natural language description of the content in an image. It lies at the intersection of computer vision and NLP. Automatic image captioning is useful for many applications, such as developing image search engines with complex natural language queries and helping the visually impaired understand their surroundings. Hence, image captioning has been an active research area. The advent of new CNNs and object detection architectures have contributed enormously to improving image captioning. Moreover, sophisticated sequential models, such as attention-based RNNs, have also been presented for accurate image caption generation.

#### VQA

Visual Question Answering (VQA) is a technology used for questioning and answering images (Lowe, 2004; Huang et al., 2019). The model needs to understand the basis of the image and answer the question. VQA is much more difficult and complex than image description because there are often graphs. There is no information in the image. Malinowski and Fritz (2014) are two of the first researchers to propose open-domain VQA, and also to provide a dataset for others to use for research purposes; the system can respond to simple questions, such as quantity and object. Wu et al. (2016a) proposed VQA that references external knowledge bases, which can be supplemented in detail. The architecture diagram is as follows:

1. Firstly classify the pictures by multi-label.
2. Input the first five image labels into the knowledge base to extract the external knowledge base and perform Doc2Vec decoding.
3. Generate multiple image descriptions from image tags and encode them.
4. Input the three into a Seq2Seq model at the same time as the initial state, then encode the question and decode the final answer.

### AR Applications

Zheng et al. (2020) used a wearable AR device combined with a binocular camera, with a deep learning and feature extraction to achieve a smart cable assembly system. The overall manufacturing process and the feature extraction element uses Edge detection; the binocular camera is used to predict the depth. After capturing the ROI, the assembly logic is judged according to the hole and center position. The VGG16 model is used to read the text of the cable line, which is read by the wearable AR camera and sent to the computer. It is then shown on the helmet.

## Research Methods

Although Industry 4.0 emphasizes intelligent manufacturing technology, many manufacturing scenarios still cannot be completely independent of personnel and craftsmanship. However, the number of personnel with technical ability and experience is rapidly decreasing, therefore, the knowledge and skills that will guide the work of personnel in the future will be greatly reduced. This shortage of experience must be dealt with in advance. In particular, assemblers in the manufacturing industry are essential, and newcomers will inevitably make mistakes, however, the cost of making mistakes must be minimized. Therefore, it is necessary to explore the immediate guidance or effective notification of operational knowledge at work. Integrated into this research is the use of feature extraction algorithms to enhance the image recognition capability of AR. Technologies, such as speech recognition and NLP, are also used to develop a chatbot that can interact verbally with operational knowledge and the aforementioned AR function, prompting workers in real-time to reduce the difficulty of on-site assembly tasks.

### Methods

**Figure 5:**
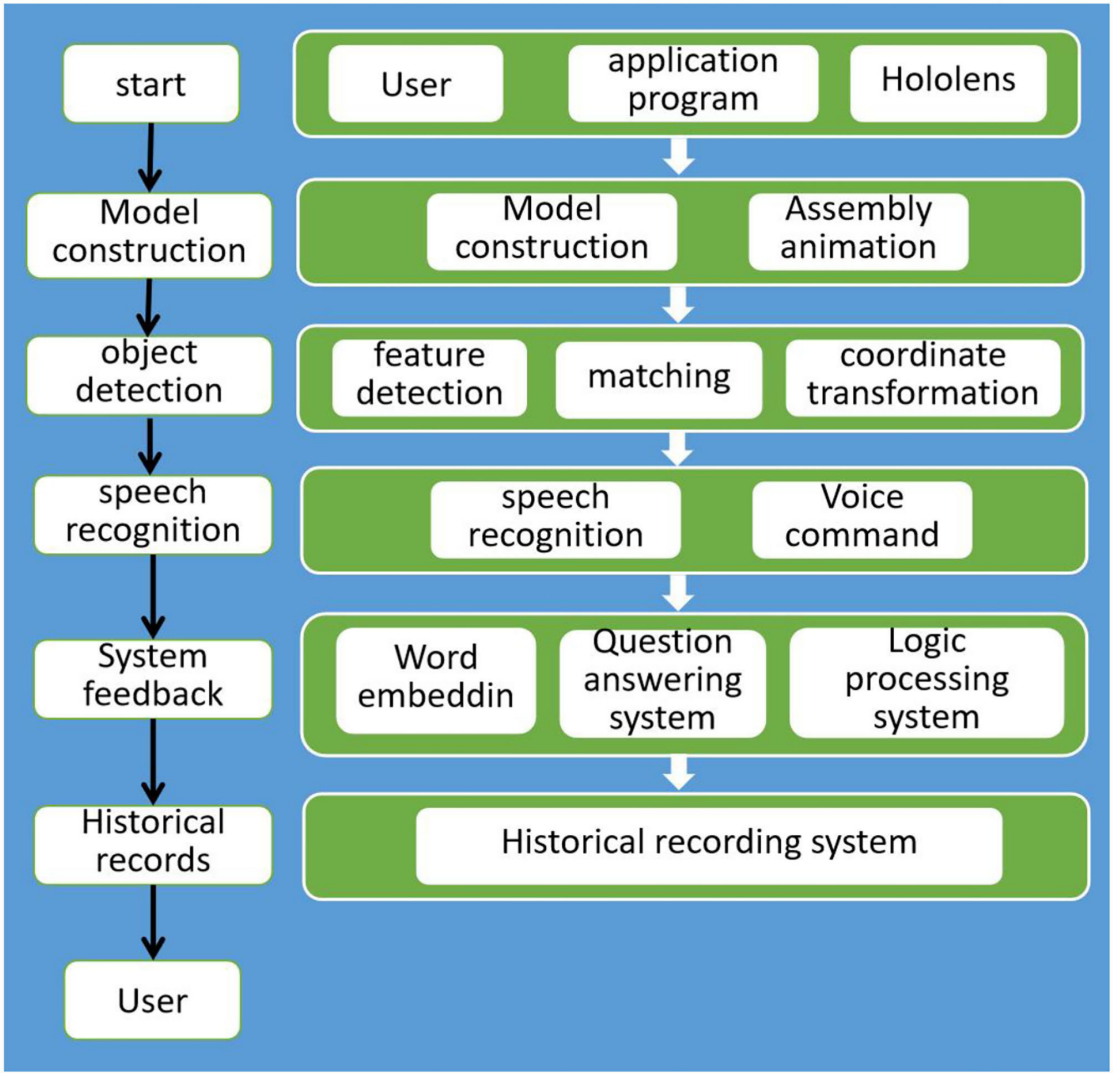
Overview of the system architecture.

This research will design animations of key assembly actions based on the assembly sequence of the manufacturing process assemblies and will allow users to use voice commands to perform steps or to ask questions of the system for additional knowledge. The application device uses Microsoft's HoloLens2HoloLens22, the purpose of which is to make the hands work easily, and the work instructions only need to be visually viewed through AR. The research process is shown in Figure 5 and can be divided into five steps:

Step 1. Model construction: in this step, the 3D model of the entity will be constructed, and the assembly animation of the 3D model will be designed according to the standard operation process of assembly, so as to fit the real entity in an intuitive way.
Step 2. Target detection: the feature points of the image are captured by the feature algorithm and comparison and matching are carried out. After the camera coordinates the transformation, the assembly animation of the 3D model is displayed on the monitor.
Step 3. Voice recognition: through voice commands, the user interacts with the system, and through automatic voice recognition technology, the audio is converted into text, so as to achieve the function of the user asking the system.
Step 4. System feedback: a logic system is designed that can set the state of the assembly stage, receive functions triggered by voice commands and receive questions from users. NLP technology is used to select appropriate answers. This is the center of the system.
Step 5. History record: during the running process of the program, the time spent in each step will be recorded and the questions asked at each stage will also be recorded. A database system will be established to organize the data. In the future, users will be able to be understood through analysis. There is a lack of knowledge at every stage.

### Model Construction

**Figure 6:**
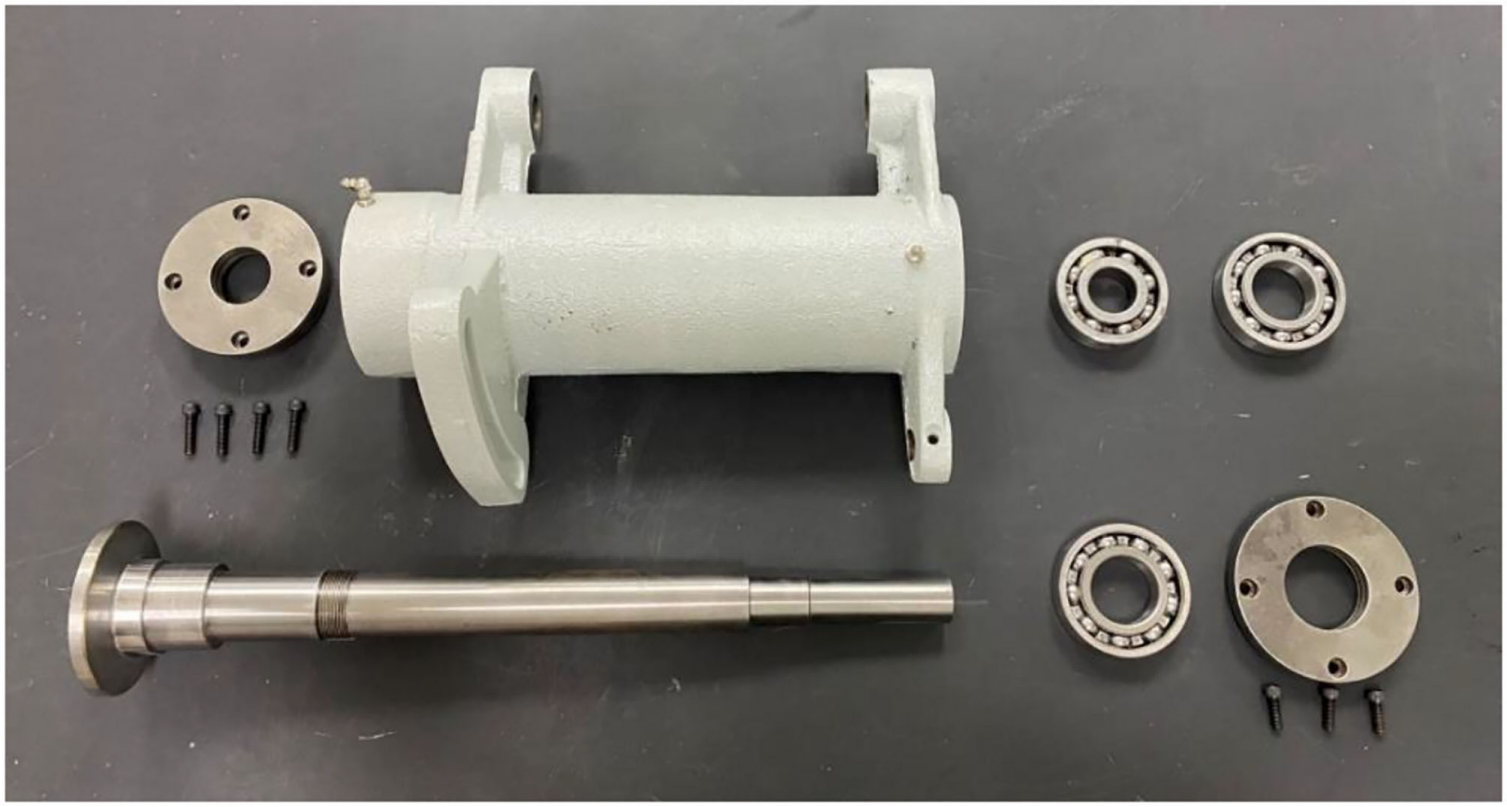
Solid assembly components.

**Figure 7:**
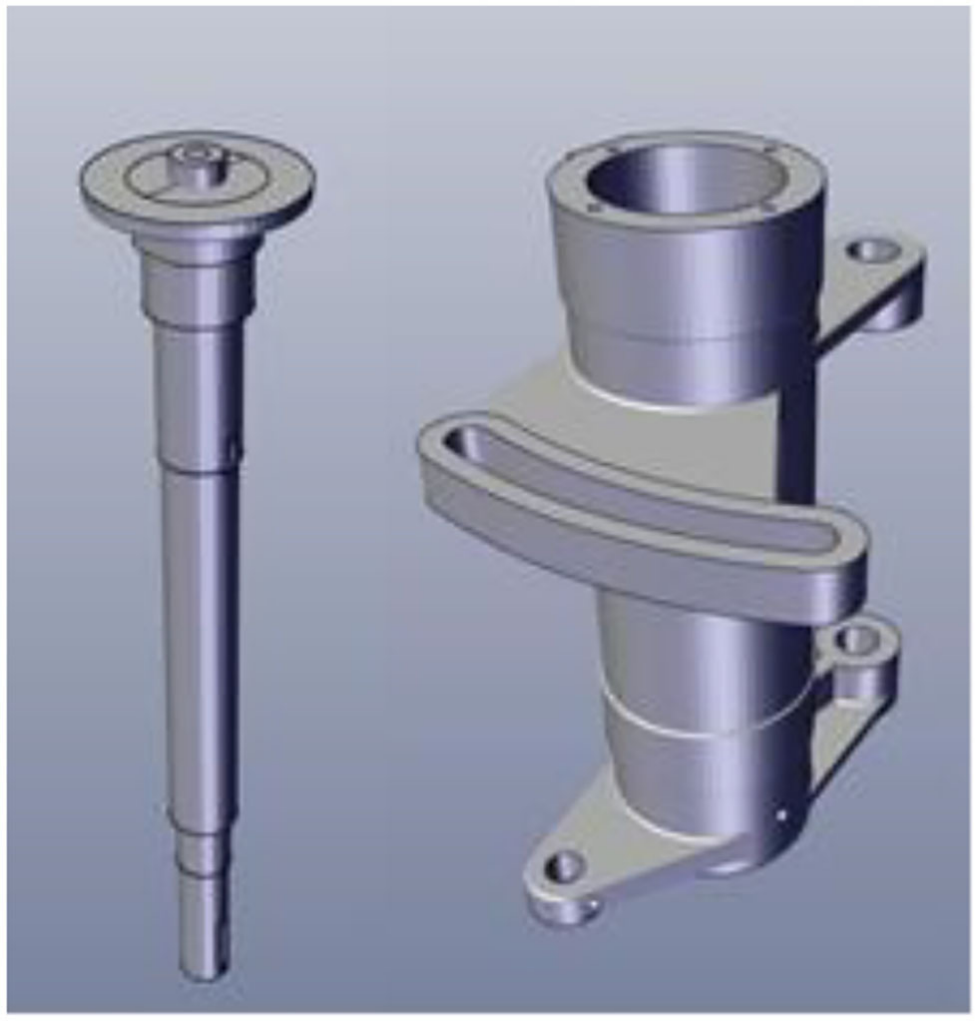
3D model of spindle and spindle housing.

**Figure 8:**
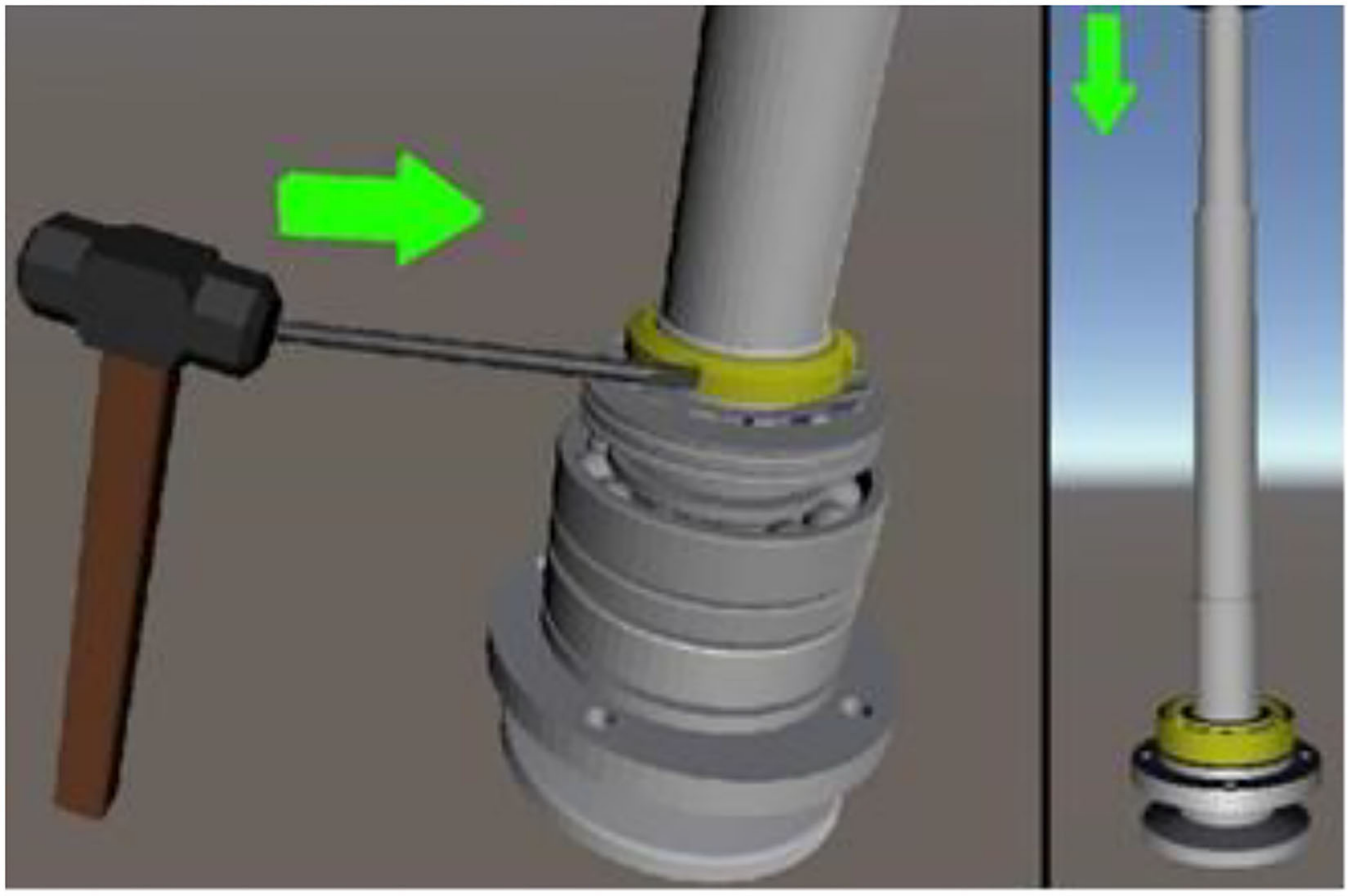
SOP animation design.

3D models have laid the foundations for many fields, for example, non-real species use 3D models. In the game field, all 3D game scenes, characters, and NPCs are also 3D models. The same is true with regard to the characters that appear in the environment. All technologies related to the 3D field are expressed based on 3D models. This study will take the manual assembly of the mechanical components to use as an example for illustration purposes. During the research, CATIA software will be used to construct a 3D model, drawn according to a 1:1 scale and then imported into the unity program for assembly animation design, so that the 1:1 model can be presented in the AR. Figure 6 shows the actual spindle group to be assembled. The 3D model is shown in Figure 7, and the animation teaching made according to the SOP process is shown in Figure 8.

### Object Detection

There is already a very mature package, Vuforia, in the field of object recognition. This tool provides various API functions conveniently and the recognition ability is also very good. However, since the application of this package in business requires authorization, an algorithm with high real-time and high recognition ability is proposed here. This is Oriented FAST and Rotated BRIEF (ORB) (Rublee et al., 2011; Shao et al., 2018). When the human eye observes an image, it is easy to determine all the corners of the image, but when the computer needs to recognize an image, this can only be achieved by calculation, therefore, a corner algorithm exists, which is a kind of feature point. The calculation steps of the Features from the Accelerated Segment Test (FAST) are as follows:

1. Given that pixel, p, is the center, there are 16 pixels p1–p1 6 in the center of the circle with a radius of 3.
2. Define a threshold and calculate the pixel difference between p1, p5, p9, p1, and the center, p. If there are more than three points that exceed the threshold, these will be regarded as candidate corners for the next step. If there are none, they are not corners.
3. Calculate the pixel difference between p1 to p16 and the center point, p. If at least nine consecutive points exceed the threshold, it is a corner point, if not, it is not a corner point.
4. Perform non-maximum suppression on the image: the sum of the absolute values of the difference between the 16 points and the center is the score value. Cut off the adjacent matrix area of the corner center, p, such as 3 × 3 or 5 × 5; if there are multiple corners, calculate them.

The score value of each feature point is found; if p is the highest score among all the corner points in the adjacent area, it is reserved, if not, it is suppressed. If there is only one corner point in the adjacent area, it is reserved and each point found is calculated by FAST corner points; these are the feature points. Since the FAST feature does not have the characteristics of rotation invariance and scale invariance, this will cause the image to be unable to match the corresponding point after scaling. To achieve scale invariance, the ORB algorithm uses a multi-scale image pyramid to solve this problem; it uses the gray centroid method (Intensity Centroid) to solve the problem of rotation invariance. The multi-scale pyramid is used to construct features of different scales. By reducing the dimensionality of the picture and generating feature points using the above steps, the features of the picture at different scales can be obtained. The gray-scale centroid method (Intensity Centroid) makes the feature points have directions, by calculating the centroid. The direction from the feature point to the centroid is taken as the direction of the feature point, such as formula (1) and (2), m is defined as the moment, that is, the centroid, C, is calculated through the moment and I(x, y) is the gray-scale of the image. This indicates that (x, y) is a point in the neighborhood and connects the centroid and the feature point to find the angle between the line and the abscissa axis, which is the direction of the feature point.


(1) $$\begin{array}{l} m_{pq}=\underset{x,y}{\sum }x^{p}y^{q}I\left(x,y\right) \end{array}$$

(2) $$\begin{array}{l} C=\left(\begin{array}{cc} m_{10} & m_{01}\\ m_{00} & m_{00} \end{array}\right) \end{array}$$

Assuming that the coordinate of the feature point is the center, O, the centroid is C and a direction vector OC is obtained, so the direction θ of the feature point can be defined as for formula (3):


(3) $$\begin{array}{l} \theta =\text{atan}2\left(m_{01},m_{10}\right) \end{array}$$

BRIEF (Calonder et al., 2010) is a feature descriptor. Only with the feature descriptor can the matching task between images be performed. The BRIEF algorithm is a descriptor that converts vectors into binary. The principle is for each In the adjacent area of the feature point, select n pairs of pixel points pi, qi (*i* = 1, 2,…., *n*) and then compare the gray value of each pair of points; if I(pi)>I(qi), then the binary number is 1, otherwise, it will generate 0. All pairs of points are compared and n pairs of binary groups will be generated; usually, 256 is selected for n. As in formulas (4) and (5), *f nd* is a BRIEF descriptor composed of n pairs of binary groups.


(4) $$\tau (\text{p};\text{x},\text{y}):=\{\begin{array}{c} 1\;ifp(x)<p(y)\\ 0\;else \end{array}$$

(5) $$\begin{array}{l} f_{n_{a}}\left(p\right):=\underset{i\leq i\leq n_{d}}{\sum }2^{i-1}\tau \left(p;x_{i}y_{i}\right) \end{array}$$

Since the BRIEF descriptor does not have rotation invariance, if the image is lost after rotation, ORB can use the previously obtained direction information, therefore, steered BRIEF was born. The original n pairs of pixels are input into the two-dimensional matrix S and are finally improved. The formula of the following brief is as formula (9); S is the set of n pairs of points, such as formula (6), θ is the direction calculated by the formula (3), R_θ_ is the corresponding rotation matrix, such as formula (7). To add rotation invariant feature descriptors. Formula (8) *S*_θ_ is calculated by *R*_θ_ plus *S*.


(6) $$\begin{array}{l} S=\left(\begin{array}{cc} x_{1}\cdots & x_{n}\\ y_{1}\cdots & y_{n} \end{array}\right) \end{array}$$

(7) $$\begin{array}{l} R_{\theta }=\left[\begin{array}{c} cos\theta -\sin\theta \\ \sin\theta +cos\theta \end{array}\right] \end{array}$$

(8) $$\begin{array}{l} {\text{S}}_{\theta }=R_{\theta }\text{S} \end{array}$$

(9) $$\begin{array}{l} g\text{n}\left(\text{p},\theta \right):=\text{fn}\left(\text{p}\right)|\left(\text{xi},\text{yi}\right)\in \text{S}\theta \end{array}$$

Using the feature descriptor, we will create a feature matcher using OpenCV's FLANN library, where the matching part uses the nearest neighbor search matching (KNN) + Lowe's algorithm; KNN matching in OpenCV is a brute force matching method, the principle of which is to match the feature points of the matching map with the matching points of the target image to find the closest top K. Lowe's algorithm selects a feature point on an image and finds the first two key points with the closest Euclidean distance in the image to be matched. Among the two key points, the closest distance is divided by the second closest distance to obtain a ratio less than defined, if the threshold value, T is T, then this pair of matching points is accepted and the ratio is usually set to 0.6. The homograph matrix, which seeks the matrix of an object from one perspective to another perspective transformation matrix H, is defined as for formula (10):


(10) $$\begin{array}{l} H=\left[\begin{array}{ccc} h_{11} & h_{12} & h_{13}\\ h_{21} & h_{22} & h_{23}\\ h_{31} & h_{32} & 1 \end{array}\right] \end{array}$$

The conversion relation is as in formula (11); *x* ′ and *y*′ represent the coordinates of another view:


(11) $$\begin{array}{c} \left[\begin{array}{c} x^{\prime }\\ y^{\prime }\\ 1 \end{array}]=[\begin{array}{ccc} h_{11} & h_{12} & h_{13}\\ h_{21} & h_{22} & h_{23}\\ h_{31} & h_{32} & 1 \end{array}][\begin{array}{c} x\\ y\\ 1 \end{array}\right] \end{array}$$

Therefore, to recover the eight parameters of H, at least four equations are required, that is, four sets of matching points, and the above formula can be expanded to formula (12):


(12) $$\begin{array}{c} x^{\prime }=\begin{array}{c} h_{11}x+h_{12}y+h_{13}\\ h_{31}x+h_{32}y+h_{33} \end{array}y^{\prime }=\begin{array}{c} h_{21}x+h_{22}y+h_{23}\\ h_{31}x+h_{32}y+h_{33} \end{array} \end{array}$$

Expand again to get formula (13):


(13) $$\begin{array}{c} \begin{array}{c} h_{11}x+h_{12}y+h_{13}-h_{31}xx^{\prime }-h_{32}yx^{\prime }-h_{33}x^{\prime }=0\\ h_{21}x+h_{22}y+h_{23}-h_{31}xy^{\prime }-h_{32}yy^{\prime }-h_{33}y^{\prime }=0 \end{array} \end{array}$$

Assuming that N matching point pairs in the two pictures are obtained, the formula (14) can be obtained as follows:


(14) $$\begin{array}{c} \begin{array}{c} x_{1}y_{1}1000-x_{1}x_{1}{}^{\prime }-y_{1}x_{1}{}^{\prime }-x_{1}{}^{\prime }[h_{11}]\\ 000x_{1}y_{1}1-x_{1}x_{1}-y_{1}x_{1}[h_{22}]\\ 000x_{n}y_{n}1-x_{n}x_{n}{}^{\prime }-y_{n}x_{n}{}^{\prime }-x_{n}{}^{\prime }[h_{33}] \end{array} \end{array}$$

It can be seen from the formula that at least four sets of matching points are required to obtain the perspective transformation matrix H of the two views. The Random Sample Consensus (RANSAC) algorithm is an effective method for denoising the impact estimation model, which can reduce incorrect matching points. The algorithm is as follows:

1. Randomly extract four sample data from the matching point dataset and calculate the transformation matrix H.
2. Calculate the projection error between all matching points and model M in the data. If the error is less than the threshold, t, add the intra-office point as formula 15.
3. If the number of points in the government interior-point set exceeds the optimal interior-point set I_Best, then update I_Best and update the number of iterations, k, at the same time.
4. Exit when the iteration exceeds the value of K and return to the model with the largest number of intra-game points, otherwise repeat the above steps.

(15) $$\begin{array}{c} \|\begin{array}{c} x_{1}{}^{\prime }\\ y_{1}{}^{\prime }\\ 1 \end{array}-H[\begin{array}{c} x_{1}\\ y_{1}\\ 1 \end{array}]|\leq t \end{array}$$

Before adding RANSAC, there are many wrong matching points and each line is a set of matching points. After adding RANSAC, the green line is the correct matching and the black box is the correct matching mark display, which is consistent with the real environment. It is necessary to verify again that the matching is correct.

### Speech Recognition

Under the existing technology, voice control is no longer an unreachable technology, and the purpose is to facilitate instant responses and relieve the burden of the interactive input of the two-hand operation. On the interface of digital work, if sound can be used to interact steadily to achieve work, this will greatly increase efficiency. Therefore, this study will use Google Voice Service and HoloLens2HoloLens22 built-in voice commands to construct a speech recognition function, namely, the architecture. Since hands are frequently used to operate tools during work, gesture interaction is avoided as much as possible during operation. Microsoft's MixedRealityToolKit (MRTK) tool for HoloLens2HoloLens22 is used to attach voice commands to the research software. The function of Google's Speech-to-Text is designed in the form of an Application Programming Interface (API), as long as the key value, the language to be recognized, etc. are passed in through the URL. It is very convenient to upload audio files through POST and to let the system return a json format recognition result. Research can use PostMan software to help test whether the API can be run, so as to achieve the required results. It can see the ranking of the returned results and the confidence level. The higher the confidence level, the higher the identification result. In research, not only can the first value be returned as the identification result, but the identification result can also be confirmed by setting the confidence level.

### System Feedback

Since there may be multiple users operating at the same time, this project designs a system server to interact with all users. Due to the performance limitations of HoloLens2HoloLens22 itself, most of the operations are handed over to the server for calculation, and finally, the results are output to the user. The system uses the TCP/IP communication protocol to interact with the user. During the process, the user will send different request commands to the system, and the server will return the results of each task according to the different commands. The system includes several modules: 1. Conversational bots. 2. Instant status display. 3. System knowledge base.

#### Chatbot

**Figure 9:**
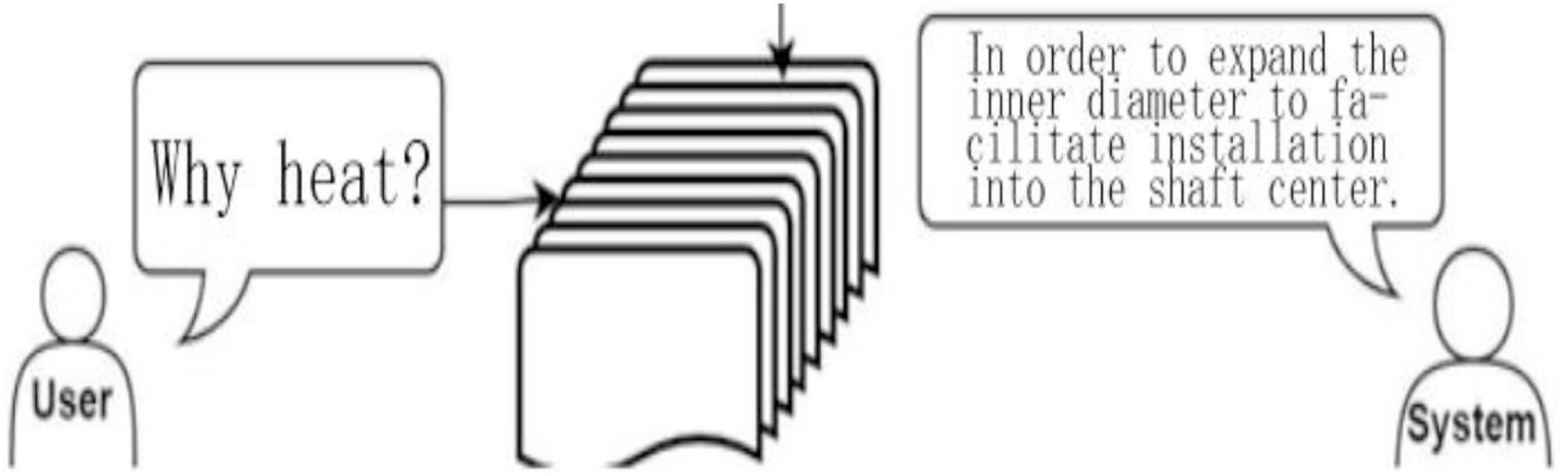
Schematic diagram of the dialogue robot.

Google speech recognition is performed according to the voice file sent back by the client, and after converting it into text, it is input into the dialogue management system to compare the data in the system knowledge base. Each project has a project document, the content of which is the knowledge contained in the project, and the dialogue robot is mainly used to capture a certain paragraph of the document for output, as shown in Figure 9; this technology is then applied to NLP. The reason for designing the reading comprehension as a closed domain is that the SOPs of most companies or factories are very different. The knowledge base is also somewhat different, and even some patent issues are involved, so we designed the robot as a closed domain. According to the corresponding document of the project, after the knowledge is returned, the subsequent new knowledge action only needs to add the knowledge content of each project.

To realize the dialogue robot, it is necessary to realize the functions of article search, reading comprehension, and speech generation, each of which is related to one other. Article search uses two technologies, BM25 and inverted index. BM25 is an improved algorithm of Term Frequency–Inverse Document Frequency (TF-IDF). TF-IDF is divided into two parts, term frequency (TF) and inverse document frequency (IDF). The word frequency represents the total number of times the keyword appears in the article. The higher the value, the greater the relevance. However, the so-called stop words must be added to the calculation. Words, such as only, like, as long as, and later, have no effect on the article. Contributed words can be deleted and the calculation of word frequency is performed. The inverse text frequency indicates the importance of a word, such as the principle of bearing. It is obvious that the importance of “bearing” is higher than that of principle. If a word appears very few times in all documents, this represents its importance. The degree is high, otherwise, it is low. The calculation method and the algorithm of TF-IDF is formula (16). The larger the value, the higher the correlation. The BM25 algorithm is an improved algorithm of TF-IDF, which is commonly used in search engines and recommendation systems. BM25 is such a formula (17), and k represents the upper limit of the value. The idea is that the influence of a keyword cannot be infinite, but there should be a maximum value, where k is pre-set to 1.2, d is the length of the article, avgeD is the average length of the article and b is the degree of influence of the length of the article on the score.


(16) $$\begin{array}{l} \text{TF}-\text{IDF}=\text{TF}\times \text{IDF} \end{array}$$

(17) BM25=IDF×{(k+1)×TF}/{k×(1.0−b+b×(d/avgD))+TF}

**Figure 10:**
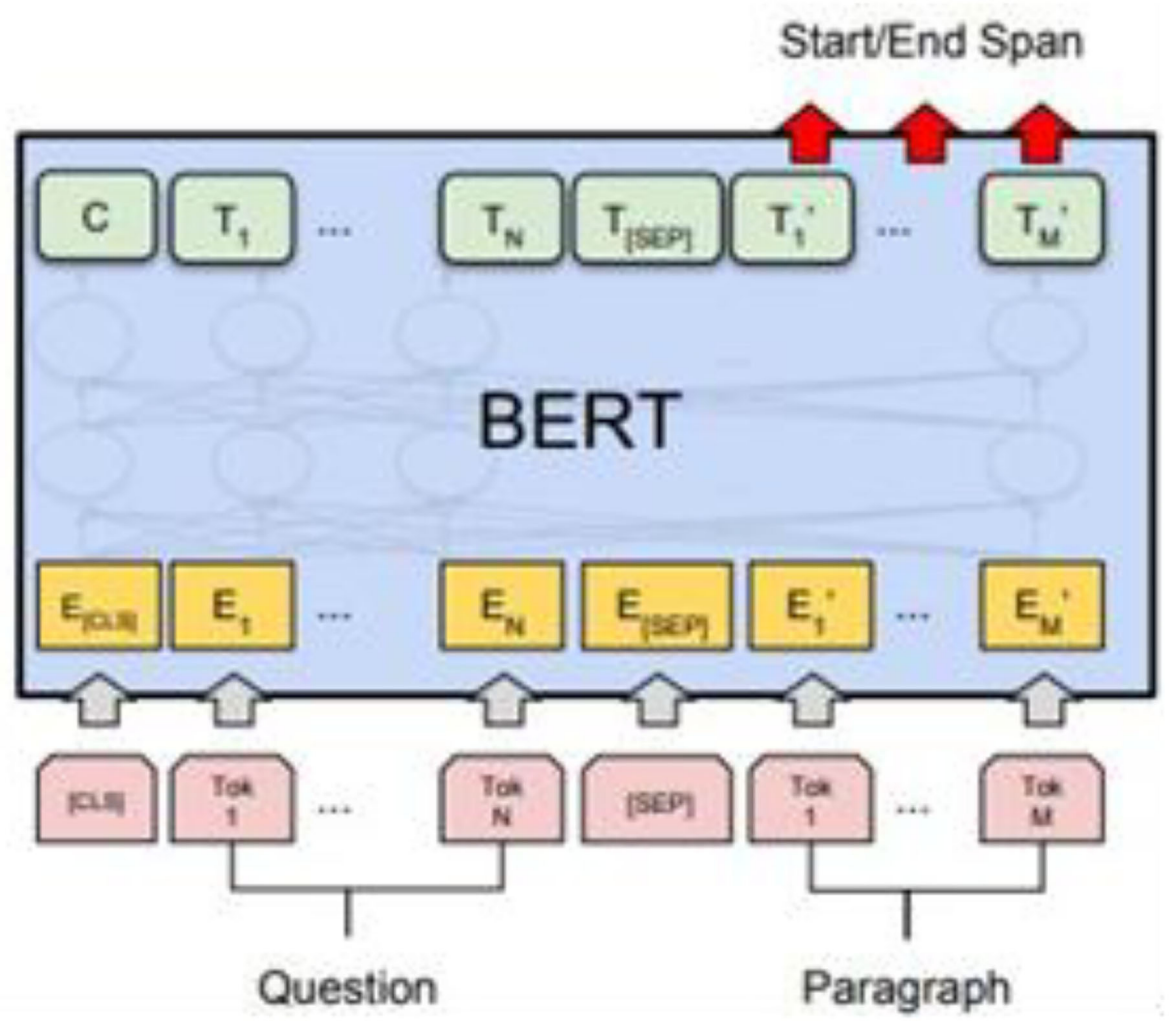
The Bert language model.

The inverted index is also known as a reverse index. The principle is to write the vocabulary and document number, as well as the number of occurrences into a dictionary structure. Finally, the documents associated with all the words of the query sentence are calculated by BM25 to obtain the total score and then sorted to obtain the document relevance, which realizes the function of article search. This module is designed in the way of API to achieve cross-platform functions so that every terminal device can access it. We build API services by using the Flask framework in the Python language. During the process, we use Json Web Token (JWT) for request verification; it is necessary to log in before using the API and to obtain a Token sent by the system. When you submit the API in Post, you must have the Token authentication before you can send a POST request. In the Header, you must add the Authorization key and value. A Token must be added after Bearer. In the system response section, we use Google's BERT model to train the Question Answering (QA) task set; here we use the Delta QA training set (Shao et al., 2018). The dataset has more than 30,000 questions marked from the wiki and the data format and the real data are viewed using Json Viewer. The BERT language model, produced by Google, is actually a pre-training model. The Chinese model “BERT-base-chinese,” trained by Google is used in the research, using 12 layers, 768 hidden units, and 12 self-attention layers. With 110 million parameters, the downstream task model, BERT for QA is based on the pre-trained model. The input is the context plus the question and the output is two integers, which are the index positions of the beginning and end of the answer, as shown in Figure 10.

With the input, there are two outputs. The answer is at the starting index position of the original text and the ending index position. Through training, we calculate our loss, as well as the evaluation indicators, Exact Match (EM). The F1 score, in the calculation of the evaluation score, has two indicators, Recall and Precision, which were used in the previous session. Positive samples and negative samples will be used here. The relevant information is shown in Table 1. The F1 score can express the quality of a model, that is, the average of the recall rate and the precision rate, as formulas (18), (19), and (20).


(18) $$\begin{array}{l} \text{Recall}=TP/\left(TP+FN\right) \end{array}$$

(19) $$\begin{array}{l} \text{Accuracy}=TP/\left(TP+FP\right) \end{array}$$

(20) $$\begin{array}{l} \text{F}1\text{Score}=2*\left\{\left(Precision*Recall\right)/\left(Precision+Recall\right)\right\} \end{array}$$

**Table 1:** Sample table.

	**Positive sample**	**Negative sample**
Prediction is true	TP (True positive)	FP (False positive)
Prediction is false	FN (False negative)	TN (True negative)

In the reading comprehension task, EM means that the calculation result completely matches the standard answer, F1 refers to the word-level matching degree between the calculation result and the standard answer, therefore, EM is also called the exact matching degree, and F1 is called the fuzzy matching degree. To achieve voice interaction, the voice generation function of Google API is used to create a robot output voice to let users know the intention, and PostMan is used to testing whether the function is normal. audioContent is the content of the audio file, read as an audio file. The project creation method uses the project name as the document name, such as giant axis group.txt. Taking institutional assembly as an example, knowledge relating to the component number, model, component function, product component version, assembly process precautions, product data management, product life cycle management, and other knowledge should be incorporated into the knowledge base.

## Implementation and Results Comparison

**Figure 11:**
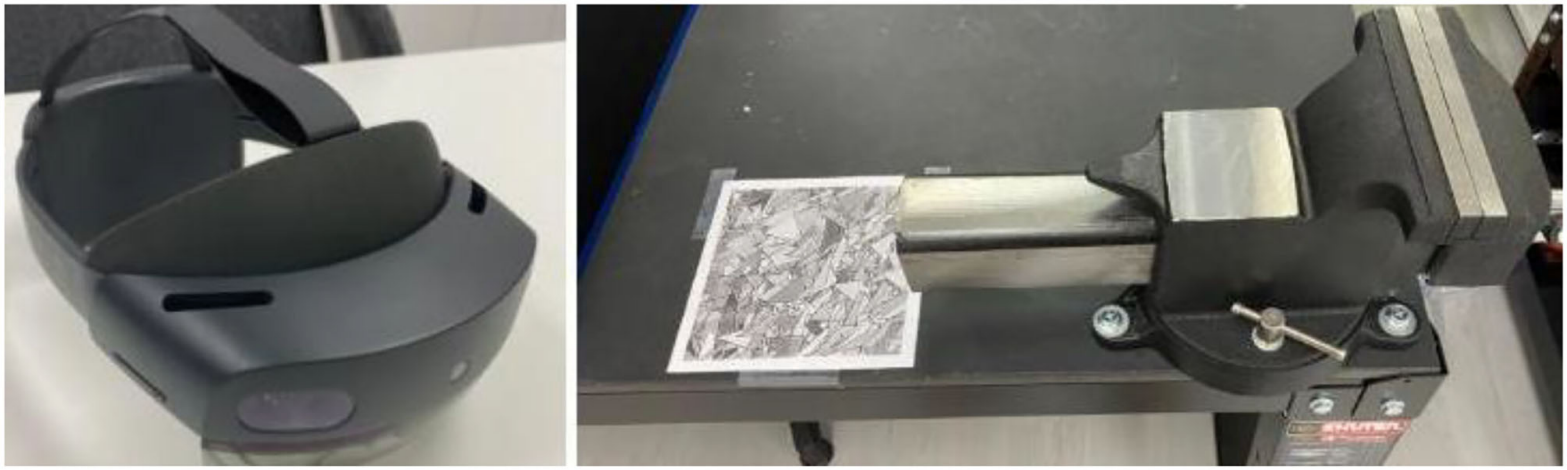
HoloLens2HoloLens222 **(Left)** and marker and vice **(Right)**.

This uses the SOP to assemble a set of giant shaft components, to test the implementation method of this study. The AR equipment used is listed in Table 2, and the environment is shown in Figure 11.

**Table 2:** Device name list.

**Device/software name**	**Quantity**	**Function**
Microsoft HoloLens2HoloLens222	1	Offers wearable AR glasses
		and AR computing
Worktable	1	Simulation assembly
Graphical Marker	1	Identify spatial positioning

The research uses the Marker to initially locate the workpiece position, then clamps the workpiece with vice and completes the display of each function item by item.

### Implementation

**Figure 12:**
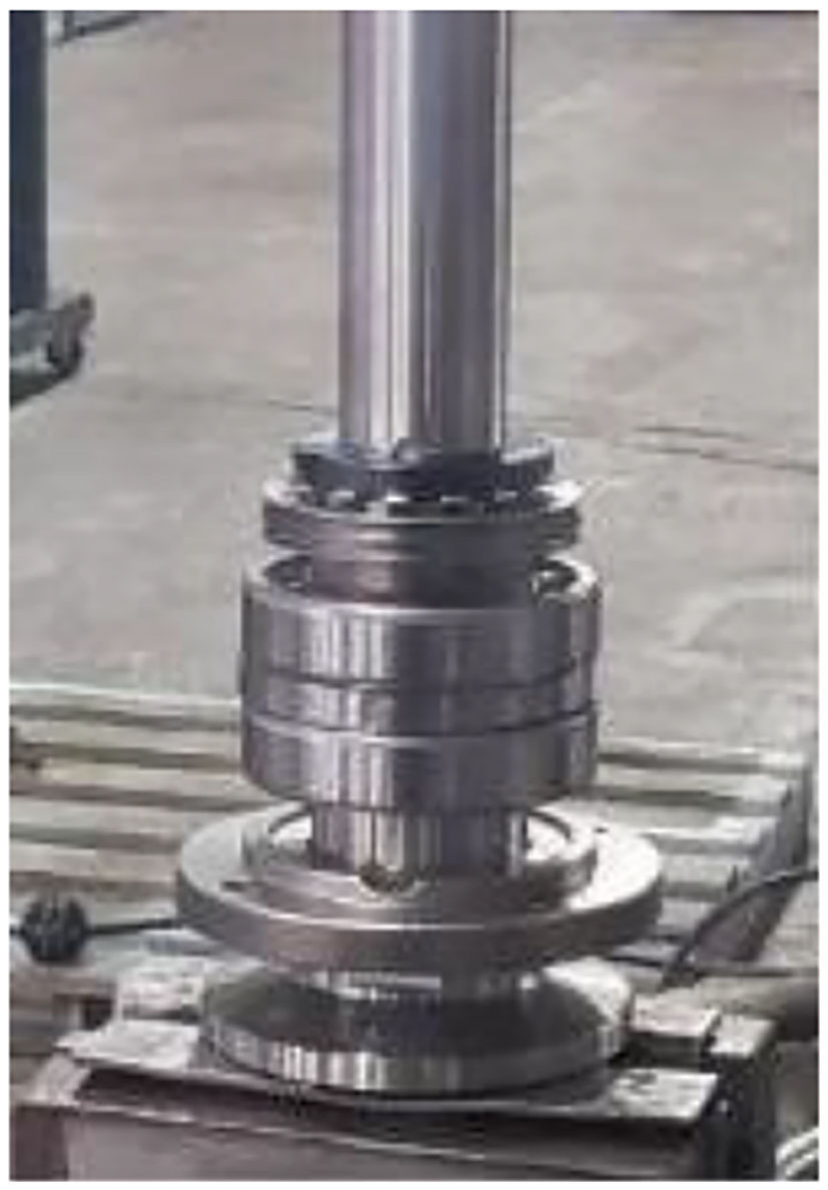
Assembled spindle.

**Figure 13:**
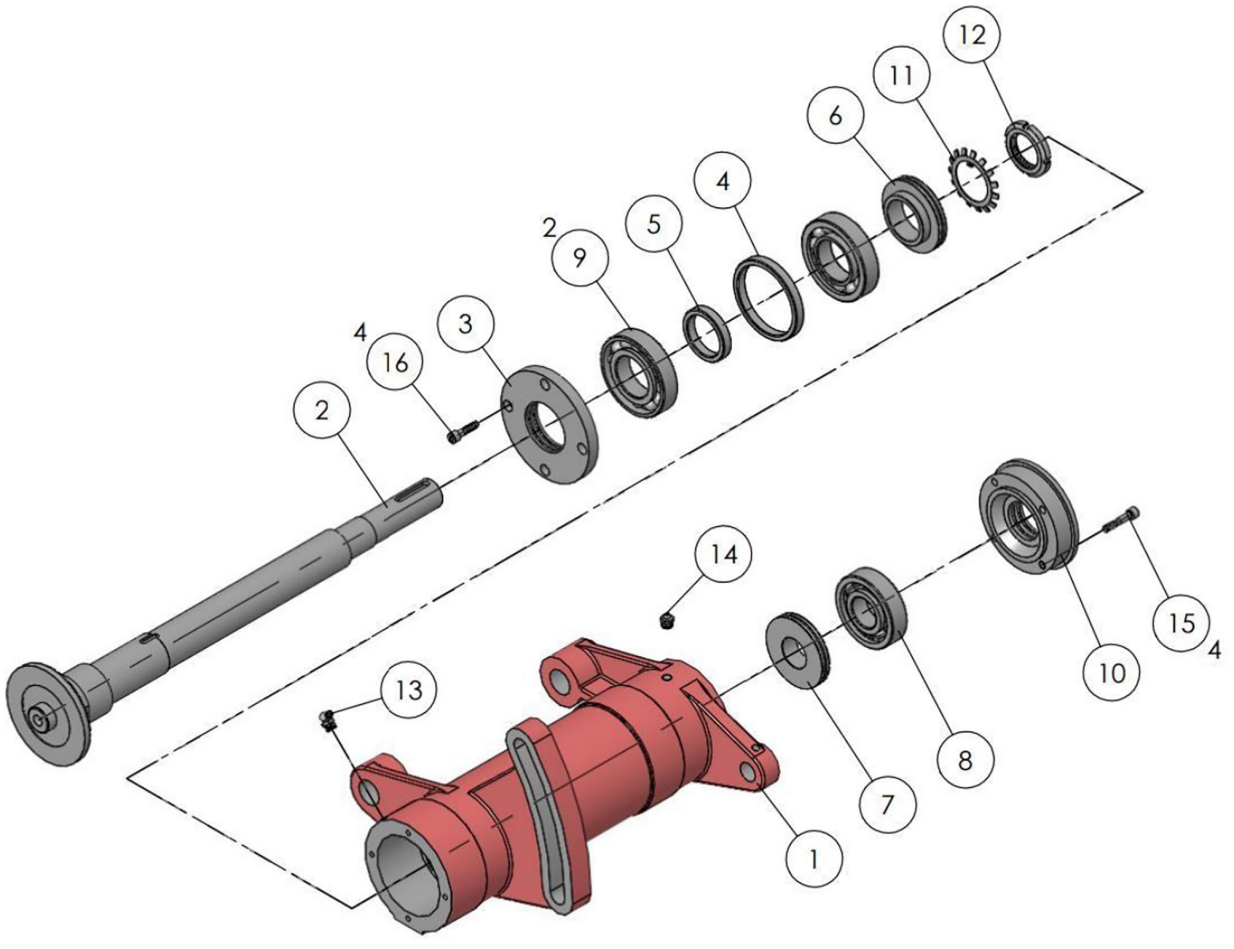
Axis assembly sequence diagram.

**Figure 14:**
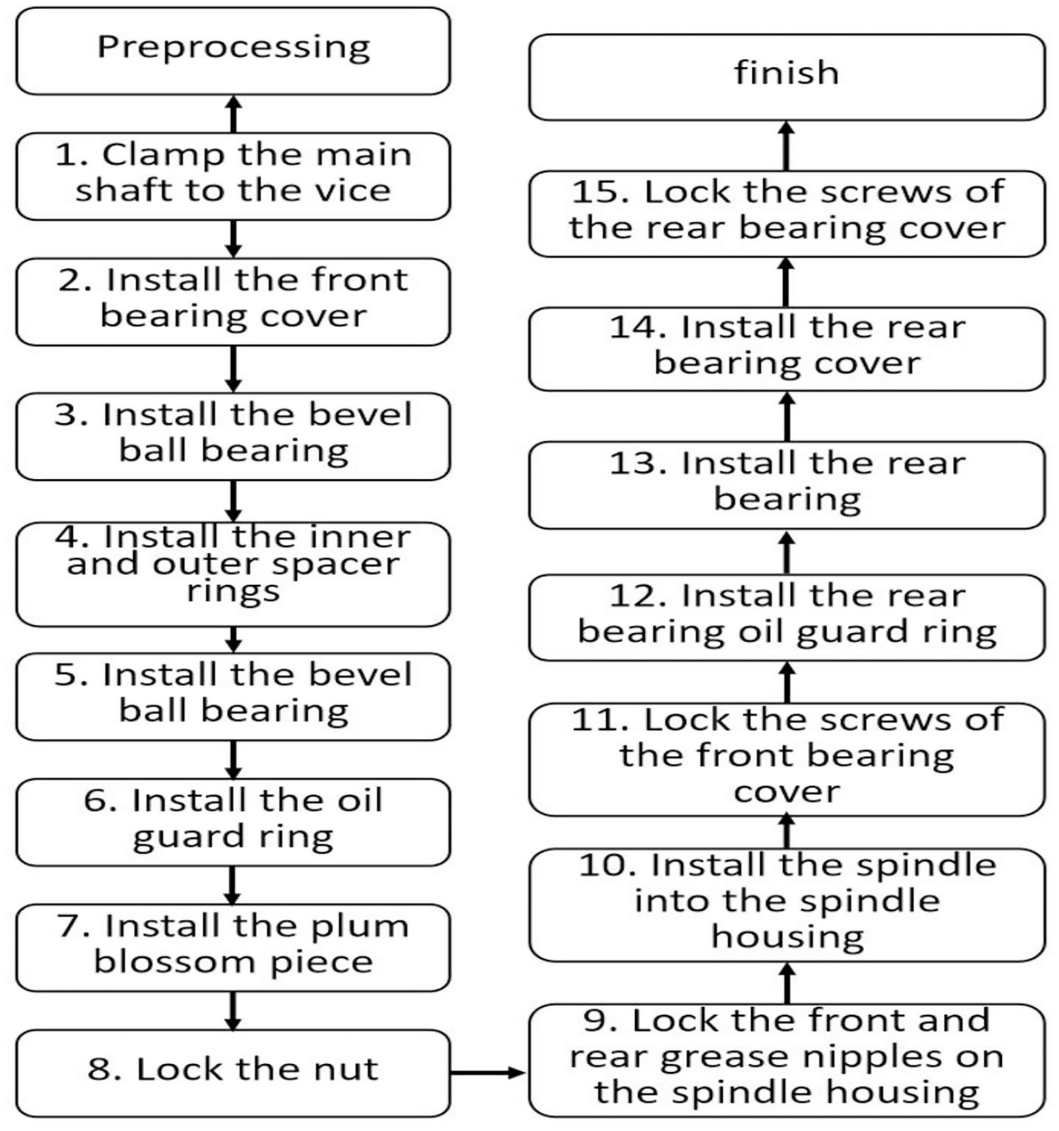
Flow chart of giant axis group SOP.

The working component of this implementation is the saw shaft group and the relevant parts and the actual appearance of the workpiece before assembly, to consider the confidentiality of the manufacturer's information. The saw shaft assembly is roughly divided into the main shaft and the main shaft housing. The assembly step is the process of sequentially loading the parts into the main shaft housing. Figure 12 shows the assembled main shaft and the photo of the finished product, with the main shaft installed in the main shaft housing. Figure 13 is a schematic diagram of the SOP assembly axis, the specific SOP process. Figure 13 is a schematic diagram of the SOP assembly axis and the specific SOP process work items are shown in Figure 14.

Among them, each step of the SOP has issues requiring attention, which are described as follows:

Step 1: First add butter to the bearing for lubrication, the dosage is around 10 cc. Then use detergent to wipe the spindle and various parts.
Step 2: When installing the front bearing cover, pay attention to the convex side facing up.
Step 3: The oblique angle ball bearing must be heated to 90–100 degrees before installation. The purpose is to expand the inner diameter so that it can be installed in the shaft. Pay attention to the installation direction, which is the same as the installation in step 5. Angled ball bearings must be installed back-to-back.
Step 4: When installing the oil retaining ring, pay attention to the convex side facing down.
Step 5: When inserting the torx slices, align the grooves.
Step 6: When installing the nut, pay attention to the processing plane facing down, then use a hammer to lock it by hand until the plum blossom piece cannot be rotated by hand, and the notch is aligned with the plum blossom piece.
Step 7: When locking the butter spout, the front end is the butter spout (90 degrees) and the rear end is the butter spout (straight).
Step 8: When installing the spindle into the spindle housing, use a heater to heat the spindle housing for 2 min.
Step 9: Use newspaper as a space between the heater and the spindle housing to prevent metal contact.
Step 10: When installing the rear bearing oil protection ring, pay attention to the convex surface facing outward.
Step 11: When installing the rear bearing, use a tool to knock the bearing into position, so that it can be accurately installed in the shaft center.
Step 12: When installing the rear bearing cap, pay attention to the convex side facing inward.
Step 14: When locking the screws of the rear bearing cover is completed.

**Figure 15:**
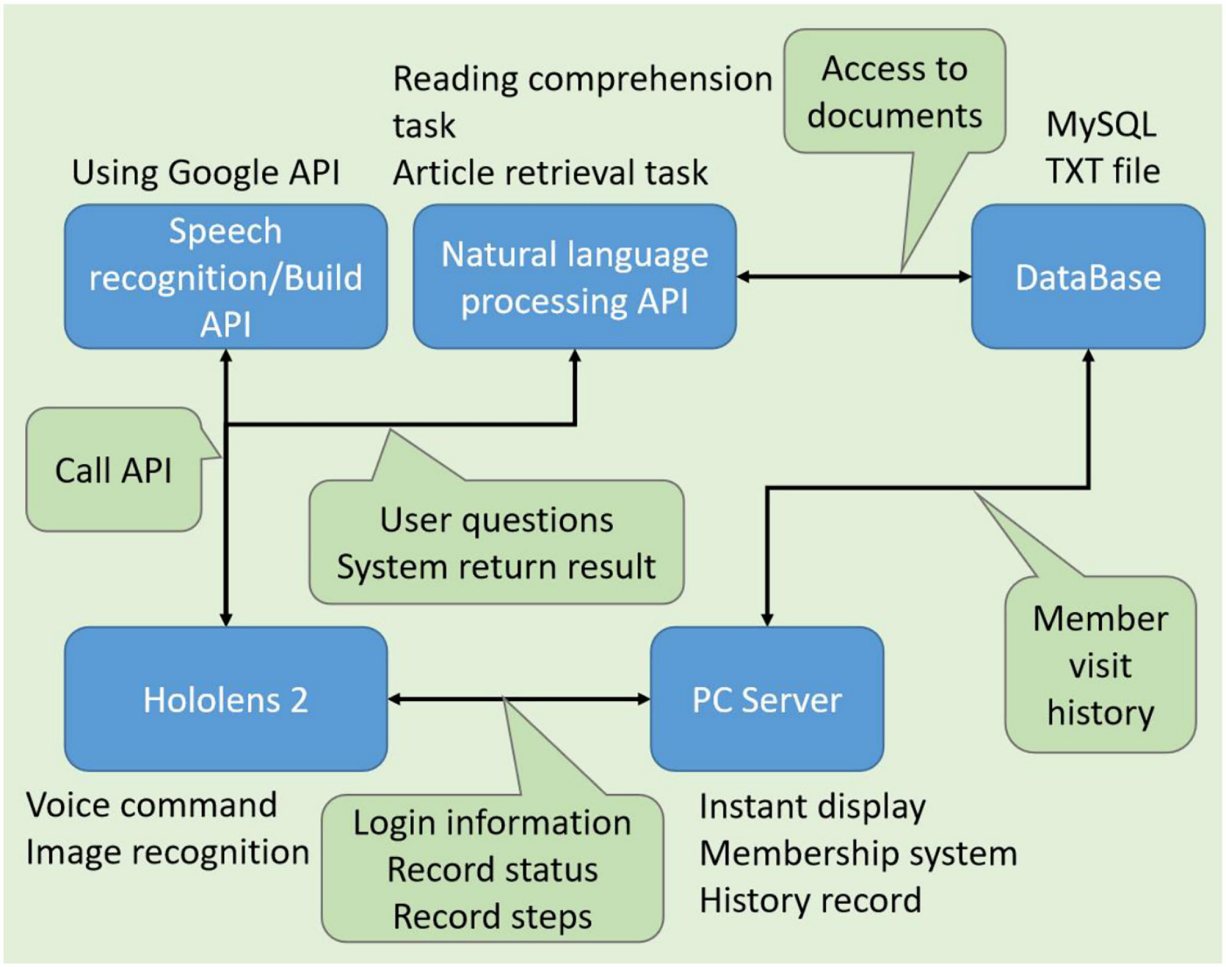
Software architecture diagram.

According to the SOP of the giant axis group, there are many details that are not mentioned in this assembly or are mentioned, but the operator does not know why this is, because each operator's knowledge is different. The equipment that has been touched and used is also different, and naturally, the experience is different. This is the difference between the old masters of those factories and the young workers. To implement the whole system, we designed the software architecture. As shown in Figure 15, the modules of each function are combined and applied to the device of HoloLens2HoloLens22. The following will use the example of this giant axis group and implement it step by step.

### Image Recognition

**Figure 16:**
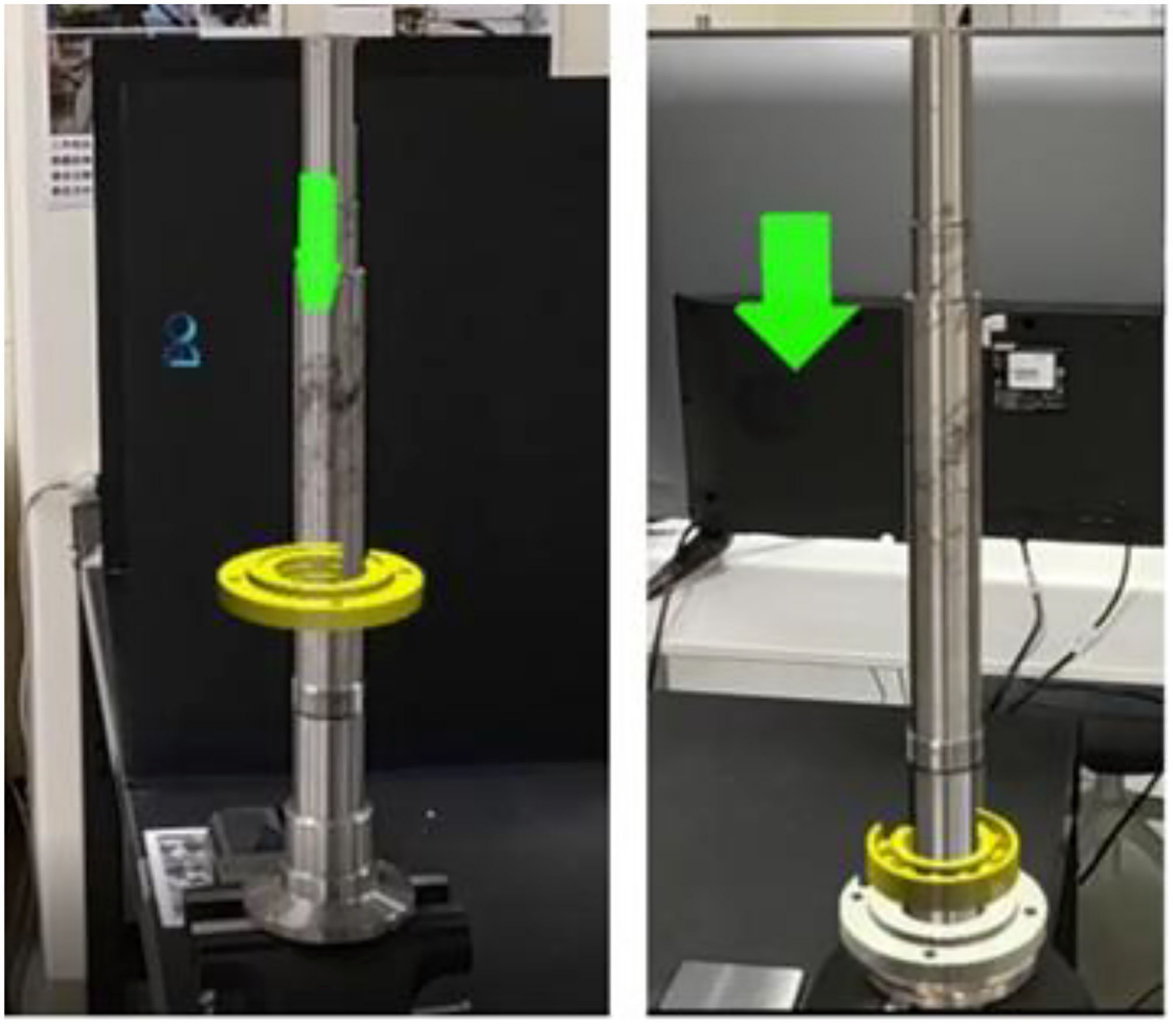
Model animation positioning.

Before each project is executed, the image recognition Marker must be used to map the position of the model to the HoloLens2HoloLens22 device, and the relationship with the real space can be located by identifying the Marker. For example, the Marker next to the vise needs to be identified before the saw axis group starts. After identification, a red frame will be displayed, indicating that the program has successfully identified the spatial orientation of the Marker. This method uses the OR B algorithm mentioned, and the average FPS is around 20. When the Marker is successfully identified, the 3D virtual model can be fused and presented on the correct line of sight screen position through its spatial positioning, providing a fully realistic visual experience. This is shown in Figure 16, using HoloLens2HoloLens22 to show the actual image captured by the screenshot function, indicating that the 3D model animation fits the physical component.

### Speech Recognition

The pre-operation of this method is a wake-up command, that is, a voice command. This method is completed through the MRTK suite of HoloLens2HoloLens22. After setting, the voice command, HoloLens2HoloLens22, can be used to trigger the start of the recording, which occurs after the voice command is reached. Next, the greenhorn pops up, intuitively reminding the operator that the voice inquiry can be performed, and the recording will be terminated after the system detects that there is no audio input for 0.5 s. In the pre-processing element of the giant shaft group, the operator may not understand why the bevel ball bearing needs to be heated, and why it can be installed after heating. At this time, the voice function can be used to query the system. When the query is completed, the audio file is uploaded to GoogleAPI to wait for the return result. The return result will be displayed on the screen, allowing the operator to confirm whether the recognition result is correct and to avoid possible recognition errors, as these may cause the system to respond differently.

### NLP API

**Figure 17:**
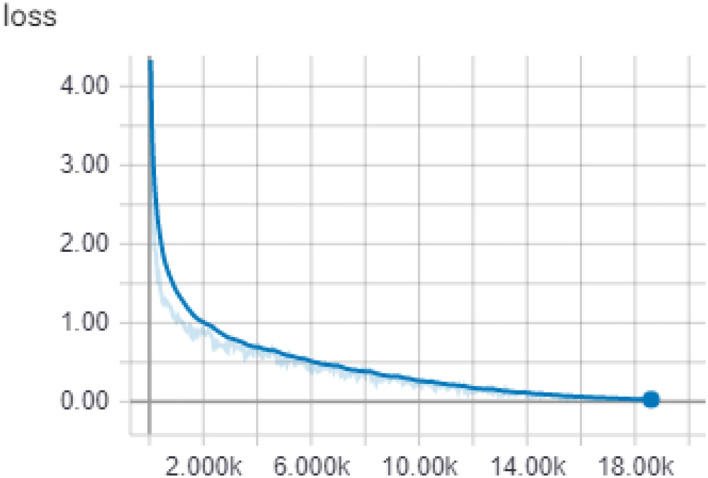
Loss score curve.

When general Internet users search for knowledge, they will first make use of various keywords to identify certain search engines, but they may also get answers of high or low relevance to narrow the scope. However, the working knowledge inquiry in this topic asks questions for a specific scope of a specific project, and the framework of this kind of inquiry must be divided into two modes when designing an inquiry system: closed domain and development domain mode. Usually, manufacturers will first record the key knowledge in a certain process of the SOP. This knowledge may be linked to company secrets, so these will not be searched in the open field. This is the default closed field mode. In the closed field, the document query method is adopted, that is, some documents are provided in advance and when the operator makes an inquiry, the system finds the appropriate answer fragment from the existing document and returns it to the user. This is machine learning, associated with the domain of reading comprehension and the training validation of reading comprehension using DRUD's dataset. The parameters of the model are shown in Table 3, which lists the parameter values that most affect the quality of the model. The final training loss score is shown in Figure 17, although many depth learning models can use the loss to understand the accuracy of the model. However, this study uses EM and F1 scores that can more clearly express the quality of the model. The number of training steps is 18,600, and the scoring index. This table shows the quality of the model. It can be seen that the EM score and the F1 score are more than half (50%), which is a good score for the reading comprehension task.

**Table 3:** Model training parameter table.

**Parameter**	**Value**
Learning rate	3e-5
Epoch	2
Batch size	32
Warm Up rate	0.1

Users can ask the system questions and can understand the responses through the document. A good case is when the query content is related to the result of the system's response, and a bad case is when the query content is not related to the result of the system's response. If the system compares the text carefully, it will find that the question and answer are related. The open domain model can be used again, that is, using article search technology, similar to the function of a search engine, so as to search for channels other than the default knowledge. Firstly, the most relevant questions are listed, then the reading comprehension task is carried out. The format of the returned results is the article ID and the relevance of the query question to the article. It can be seen that the query question is related to the article ID of the resulting output.

## Discussion

In the above implementation, this research combines the contents of various related fields and applies it to the equipment of HoloLens2HoloLens22, so that the field operators can provide intuitive cognition of visual images through AR. Voice questions and answers to make up for inadequate knowledge and experience are integrated into standard operating procedures to solve specific assembly problems. On the whole, there is already an interactive process, similar to the experience of on-site guidance, but there is still room for improvement in technology. For example, the image recognition function currently uses Marker recognition, but it can be changed to not use Marker and only use the outline of the real scene object. The spatial coordinates of the model can be quickly positioned by taking pictures, which will be more convenient for the implementation process. Compared with the traditional method, as shown in Table 4, the two are similar in time, but there are differences in continuity. Using the traditional paper version of the SOP may be difficult for operators to understand the description, therefore, the continuity of the work is not sufficient in terms of the success rate of this research. According to the research, the SOP of auxiliary wisdom teaching is stable and high, because it has a more intuitive and easy-to-understand nature. The traditional paper-based SOP has low returnability, and it is almost impossible to understand and know the current operation situation of the user. It can only reply using the oral method, but the smart teaching SOP can read and record the process through the digital environment, so as to understand the process in real-time. Various situations of operation at that time can result in an intelligent response.

**Table 4:** Comparison between traditional paper version and this research method.

	**Traditional paper**	**Smart teaching**
	**SOP**	**SOP**
Time efficiency	Low	Low
Continuity	Medium	High
Success rate	Medium	High
Returnability	Low	High
Ease of remote communication	Low	High
Custom edition convenience	Low	High

## Conclusion

Although manufacturing technology has developed to the level of control by machine intelligence, many manual operations still exist in the delicate assembly or warranty process, and these operations require more professional knowledge and experience to back up. Regarding the increasingly serious situation of low birth rates, the threshold of competence is relatively higher. However, a more serious problem is that the number of experienced workers is declining, but intuitive experience and knowledge extended by experience cannot be effectively passed on. In this regard, the research uses the highly intuitive visual interaction nature of AR and attempts to guide workers step by step in the standard process of manual work with virtual images, by providing a voice response system and way of questioning, so as to interact with standard procedures and capture relevant knowledge. Its advantages are immediate and it provides intuitive assistance that is close to the work content but also avoids the common head-mounted AR operation. The advantages of interactive support work are as convenient as those who have experience of teaching on the spot. Therefore, the research involves the recognition of images and speech, which must be fully integrated into the manufacturing process, to give the correct responses and complete the work.

### Research Contribution

This study applied AR combined with object recognition and other technologies to the intelligent assembly operation interface of institutional components, which became the SOP for intelligent interactive services. This interface can intuitively adapt the images seen by the operator's line of sight and provide users with immediate reference. If the user is not clear about the content, the system will update the obtained keywords to search for more similar information. Repeating this step will help the user to make use of the image and voice interaction to assist in completing the task, thereby supplementing the user's lack of knowledge and experience, maintaining work efficiency and quality, and synchronizing work history information, which has become a critical human-machine collaboration interface in smart manufacturing factories. In addition, since Microsoft HoloLens2HoloLens22 is used as the head-mounted AR interface in the research, should there be a requirement to develop any customized application, it will be necessary to refer to the open code and functions provided by the original factory. In this research, limited official open resources and currently available free data for editing purposes were used to gain good results. Therefore, the specific research contributions can be identified as follows: 1. the use of a feature algorithm that does not require authorization and can be optimized and changed at will. 2. this is combined with AR and integrated for HoloLens2HoloLens22 function development, completing the assembly function teaching implementation.

### Future Work

The structure of this research is not perfect, and there is still a need for improvement. In the future, if object recognition and model space positioning can be performed without using markers, the efficiency and convenience will be greatly increased. In NLP, reading comprehension will be improved. The score of the task can be higher, and the number of article documents in the article search can be increased so that the searchability and accuracy can be enhanced.

### Limitation

The object recognition technology used in this study is limited to the feature algorithm in the 2D recognition part. Only the feasible integration technologies of HoloLens2HoloLens22 2 and Unity software are discussed, and the technologies required for other AR devices are not within the scope of this study. For the reading comprehension task in this study, only the BERT pre-training model is used, and other pre-training models are not within the scope of this study.

## Data Availability Statement

The original contributions presented in the study are included in the article/supplementary material, further inquiries can be directed to the corresponding author.

## Author Contributions

ML: conceptualization, methodology, validation, investigation, writing, funding acquisition, formal analysis, software, resources, and visualization. YC: funding acquisition, methodology, validation, writing—review and editing, and supervision. Both authors contributed to the article and approved the submitted version.

## Funding

This work was supported by Key projects of social science and technology development in Dongguan under Grant (No. 2020507156156), in part by Special fund for Dongguan's Rural Revitalization Strategy in 2021 (No. 20211800400102), in part by Dongguan special commissioner project (No. 20211800500182), and in part by Guangdong-Dongguan Joint fund for Basic and Applied Research of Guangdong Province (No. 2020A1515110162).

## Conflict of Interest

The authors declare that the research was conducted in the absence of any commercial or financial relationships that could be construed as a potential conflict of interest.

## Publisher's Note

All claims expressed in this article are solely those of the authors and do not necessarily represent those of their affiliated organizations, or those of the publisher, the editors and the reviewers. Any product that may be evaluated in this article, or claim that may be made by its manufacturer, is not guaranteed or endorsed by the publisher.
